# Hepa-ToxMOA: a pathway-screening method for evaluating cellular stress and hepatic metabolic-dependent toxicity of natural products

**DOI:** 10.1038/s41598-024-54634-4

**Published:** 2024-02-21

**Authors:** Se-Myo Park, Mi-Sun Choi, Soojin Kim, Hyun Jegal, Hyoung-Yun Han, Hyang Sook Chun, Sang Kyum Kim, Jung-Hwa Oh

**Affiliations:** 1 Department of Predictive Toxicology, Korea Institute of Toxicology, 141 Gajeong-ro, Yuseong-gu, 34114 Daejeon, Republic of Korea; 2 College of Pharmacy, Chungnam National University, 99 Daehak-ro, Yuseong-gu, 34131 Daejeon, Republic of Korea; 3 Department of Human and Environmental Toxicology, University of Science & Technology, 34113 Daejeon, Republic of Korea; 4 Food Toxicology Laboratory, School of Food Science and Technology, Chung-Ang University, 17546 Anseong, South Korea

**Keywords:** Drug screening, Toxicology

## Abstract

In the field of drug discovery, natural products have emerged as therapeutic agents for diseases such as cancer. However, their potential toxicity poses significant obstacles in the developing effective drug candidates. To overcome this limitation, we propose a pathway-screening method based on imaging analysis to evaluate cellular stress caused by natural products. We have established a cellular stress sensing system, named Hepa-ToxMOA, which utilizes HepG2 cells expressing green fluorescent protein (GFP) fluorescence under the control of transcription factor response elements (TREs) for transcription factors (AP1, P53, Nrf2, and NF-κB). Additionally, to augment the drug metabolic activity of the HepG2 cell line, we evaluated the cytotoxicity of 40 natural products with and without S9 fraction-based metabolic activity. Our finding revealed different activities of Hepa-ToxMOA depending on metabolic or non-metabolic activity, highlighting the involvement of specific cellular stress pathways. Our results suggest that developing a Hepa-ToxMOA system based on activity of drug metabolizing enzyme provides crucial insights into the molecular mechanisms initiating cellular stress during liver toxicity screening for natural products. The pathway-screening method addresses challenges related to the potential toxicity of natural products, advancing their translation into viable therapeutic agents.

## Introduction

Natural products are biologically active compounds and they have been recognized as valuable sources for the development of new drugs, including those for cancer treatment^1,2^. However, the isolation and structural elucidation of active ingredients from natural products require substantial effort, leading to a decrease in interest in drug discovery^3^. Moreover, the potential risks associated with natural products have raised concerns, leading the US FDA to exclude over 1500 natural products with nutritional efficacy from use due to their preclinical stage toxicity^4^. As complexes of various components, natural products can mediate various toxic mechanisms according to each component, and have physicochemical properties of various absorption spectra. Given the complexity of natural products, they can exhibit various toxic mechanisms mediated by their multiple components, and possess diverse physicochemical properties. Consequently, the structural diversity of natural products poses challenges in drug screening, including assay system reliability, high costs, compound stability, and potential exposure risks^5^. Additionally, the complexity and unknown concentrations of natural product components make it difficult to diagnose and predict toxicity accurately^6^. Herbal-induced liver injury (HILI) has also garnered attention from healthcare professionals and regulatory agencies, yet the causes and risks of HILI remain poorly understood^7^. Understanding the hepatotoxic mechanisms of toxic ingredients in complex mixtures of herbs, which often contain both beneficial and toxic components, is essential^8^.

Toxicity evaluation of natural products has traditionally focused on single molecular targets or general toxicity, but with the current trend of reducing animal model usage, there is a need for holistic approaches and alternative methods to explore the potential of natural products^9,10^. However, existing alternative toxicity evaluation methods for natural materials, which adopt conventional chemical-based assays measuring apoptosis and cell homeostasis, are not suitable for complex natural product toxicity screening and generally analyze toxicity on an individual basis rather than in complex mixtures^10,11^. Natural products can induce toxicity through various factors, such as cytokines, DNA damage, reactive oxygen species (ROS), acute stimuli, and growth factors, leading to apoptosis, cell hyperplasia, and inflammatory reactions mediated by transcriptional mediators within a secondary signaling cascade^12,13^.

In order to evaluate and predict hepatotoxicity associated with natural products, we focused on high-content screening by establishing a toxic mechanism-based reporter system using hepatocellular carcinoma (HepG2) cells. Previous research by Wink et al.^14^ involved the creation of GFP reporter cell lines derived from HepG2 cells through bacterial artificial chromosome (BAC) cloning, which enabled the identification of stress response pathways triggered by drugs using HepG2-BAC-GFP systems. In this study, we aimed to identify toxic mechanisms induced by natural products by applying a lentiviral vector targeting activator protein 1 (AP1), tumor protein P53 (P53), nuclear factor erythroid-2-related factor 2 (Nrf2), and nuclear factor kappa-light-chain-enhancer of activated B cells (NF-κB) to HepG2 cell lines. Transcription factors play a central role in regulating gene expression, orchestrating a host of cellular processes, and are associated with toxicological responses^15^. Toxicological responses can arise from diverse molecular mechanisms mediated by transcription factors, as well as mitochondrial perturbation and autophagic regulation^16^. Both mitochondrial dysfunction and autophagy play crucial roles in hepatotoxicity, and they are intricately regulated by complex protective and compensatory mechanisms. As a result, conventional assays for these responses often demonstrate limitations, exhibiting low predictive accuracy and relevance to clinical hepatotoxicity^17^. The development of reliable cell-based assays to measure transcription factor activity is a crucial technology for fast and efficient screening of cellular stress. Furthermore, these assays provide valuable insight into the intricate mechanisms of transcription factors and their implications across a wide range of toxicological events. Additionally, the evaluation of hepatotoxicity using cell lines should be relevant to humans, particularly in terms of assessing toxicity related to drug metabolism^18^. Pyrrolizidine alkaloids (Pas), heterocyclic phytotoxins naturally occurring in over 6000 plants, can generate reactive toxic metabolites through metabolic activation by CYP450s, leading to hepatotoxicity in the liver^19,20^. Rhein, an active ingredient found in the root of rhubarb, can be metabolically activated by CYP2C19, causing mitochondrial dysfunction and hepatotoxicity^21^. Recombinant enzymes, liver microsomes, and liver S9 fractions are currently employed in in vitro drug metabolism screening studies^18,22^. Among these, liver S9 fractions consist of a mixture of unfractionated microsomes and cytosol containing various drug-metabolizing enzymes. They are easier to prepare than purified microsomes^23^ and are commonly used in in vitro ADME studies investigating phase I and phase II metabolism.

In this study, we established a fluorescence-based reporter system (Hepa-ToxMOA), targeting a representative cellular stress response in HepG2 cells. Moreover, utilizing this established reporter system, we assessed and compared the toxicity of individual components of natural products based on their non-metabolic or metabolic activity. Consequently, we successfully confirmed the toxic mechanisms induced by each component by conducting a comparative analysis of GFP expression under non-metabolic or metabolic activity conditions using cellular stress-reporter cell lines. Here, we propose a drug metabolism-based toxicity mechanism monitoring system, which allows for the rapid assessment of cellular stress induced by natural products. Furthermore, we suggest that this system can be employed in the development of novel drugs derived from natural products, as it enables the investigation of interactions based on toxicity mechanisms and the prediction of HILI through early screening.

## Materials and methods

### Cells and reagents

Human hepatoma HepG2 cells were obtained from the American Type Culture Collection (ATCC) and cell culture and in vitro assays were performed as per the manufacturer’s instructions. Information on all reagents used is detailed in Supplementary Table 1, 2.

### Establishment of cellular stress sensing cell lines

A lentiviral vector was constructed using pGreenFire1™-AP1(Plasmid) + EF1-Puro and pGreenFire1™-P53-GF-EF1-Puro (Plasmid) as a backbone. AP1-EF1-Puro and P53-EF1-Puro Plasmids contain a fluorescent GFP reporter under a minimal CMV promotor with optimized repeats of AP1 and P53 sequence motif. First, the plasmid was packaged using the pPARCKH1TM Lentivector Packaging Kit according to the manufacturer’s instructions and concentrated using a centrifuge at 1500 g for 15 min at room temperature. The viral vector was concentrated using Lenti-X Concentration. Transduction was performed using a lentiviral vector concentrated in HepG2 cells, a human hepatoma cell line. Then, stable report cell lines were constructed by sorting the transformed HepG2-GFP-AP1 and HepG2-GFP-P53 cells through BD FACS Aria. HepG2-GFP-Nrf2 and HepG2-GFP-NF-κB reporter cells were established as stable transfection of HepG2 cell lines with premade ready-to-use ARE-GFP(Puro) and NF-κB-GFP(Puro) Lentivirus according to the manufacturer's instruction. ARE-GFP(puro) and NF-κB-GFP(puro) Lentivirus contains a fluorescent GFP reporter under a minimal CMV promotor with optimized repeats of antioxidant response element (ARE) and NF-κB sequence motif. Established cellular stress-reporter cell lines were cultured in Dulbecco’s Modified Eagle’s Medium (DMEM), supplemented with 10% fetal bovine serum (FBS), 1% penicillin/streptomycin (P/S), in a 37 °C incubator with 5% CO_2_.

### In vitro testing of natural compounds using cellular stress sensing cells

Four types of toxicity-reporter cells were seeded at a density of 30,000 cells/well in a 96-well plate and incubated at 37 °C and 5% CO_2_ for 24 h. Toxicity screening was performed on 40 natural products, including alkaloids, quinones, steroids, triterpenoids, xanthones, and diterpenoids. The compounds were all dissolved in dimethyl sulfoxide (DMSO) to produce a final 50 mM stock solution. In total, 40 natural products were used to treat four different types of cellular stress-reporter cell lines by serial dilution from the final concentration in DMEM (with 2% FBS and 1% P/S).

### In vitro testing of natural compounds depending on metabolic activation using S9 fraction

Four types of toxicity-reporter cells were seeded at a density of 30,000 cells/well in a 96-well plate and incubated at 37 °C and 5% CO_2_ for 24 h. After 24 h, 9.6 ml of DMEM (with 2% FBS and 1% P/S) was added to Cofactor I and filtered with a 0.45 µM syringe filter. Additionally, 2.1 ml of filtered distilled water (DW) was added to rat liver S9 and mixed well. Then, using the prepared cofactor I, the mixture was made up to 10% rat liver S9. The prepared 10% rat liver S9 was prepared to be 2% rat liver S9 using DMEM (with 2% FBS and 1% P/S). We co-treated the cell lines with 50ul of 2% rat liver S9 and 50ul of natural products (final 1% rat liver S9) for 24 h and the control was treated with 50ul of 2% rat liver S9 and 50ul of 0.1% DMSO (final 1% rat liver S9).

### Western blotting

Four types of cellular stress-reporter cell lines are seeded at a density of 500,000 cells/well in a 6-well plate and incubated at 37 °C and 5% CO_2_ for 24 h. Phorbol 12-myristate 13-acetate (PMA, HepG2-GFP-AP1), Nutlin-3 (HepG2-GFP-P53), DL-sulforaphane (HepG2-GFP-Nrf2), and TNF-a (HepG2-GFP-NF-κB) dissolved in DMSO were used as positive chemicals for each cellular stress-reporter cell line test. After treatment with the positive chemical for 24 h, protein was extracted from cells using RIPA buffer (150 mM NaCl, 20 mM Tris–HCl pH 7.4, 2 mM NaF, 2 mM EDTA, 5 mM sodium orthovanadate, 1% Triton X-100, 1 mM PMSF, protease inhibitor cocktail), and protein quantification was performed using Pierce BCA protein assay kit. Then, Anti-TurboGFP (d) antibody and Anti-EGFP antibody were used to quantitatively analyze GFP intensity according to treatment with positive chemicals using a ChemiDoc MP imaging system (Bio-Rad, CA, USA) through western blot. All antibodies were diluted to 1:1000 with 5% skim milk (with TBS buffer containing 0.05% Tween 20).

### Imaging analysis of cellular stress sensing cell lines

Cellular stress-reporter cell lines were seeded in 96-well plate and allowed to stabilize overnight, following which they were exposed to the natural products for 24 h. Typically, six control wells each for positive and negative controls were included on each plate. After removing the natural products, DPBS (with calcium chloride, magnesium chloride) containing 10 mg/ml Hoechst 33,342 and 2 mM Calcein-AM is added to the cells and incubated at 37 °C for 20 min. At the end of the reaction, after washing twice with DPBS (with calcium chloride and magnesium chloride) including 0.1% FBS according to the manufacturer’s instructions, analysis was performed using a high-content imaging system (HCS, Molecular Devices, CA, USA). Images were acquired using ImageXpress Micro XLS (Molecular Devices), with a 10 × Plan Fluor objective lens. A DAPI filter cube was used to confirm Hoechst 33,342, a FITC filter cube was used to confirm GFP expression and a Texas Red filter cube was used to confirm Calcein-AM.

### Statistical analysis of in vitro testing data

Images were analyzed using MetaXpress 6 (Molecular Devices). Count Nuclei and Cell Scoring application modules were used for the nuclear count and live/dead assessment, respectively. GFP-positive cells were counted, and their area and intensity values were recorded. Experiments were repeated at least three times and results are expressed as mean ± standard error. Two-sided *t*-test were used to analyze the statistical significance of the data. GraphPad Prism 8.0 (GraphPad, CA, USA) was used for all analyses. *P* values < 0.05 were considered statistically significant.

## Results

### Establishment of cellular stress sensing cell system (Hepa-ToxMOA)

**Figure 1:**
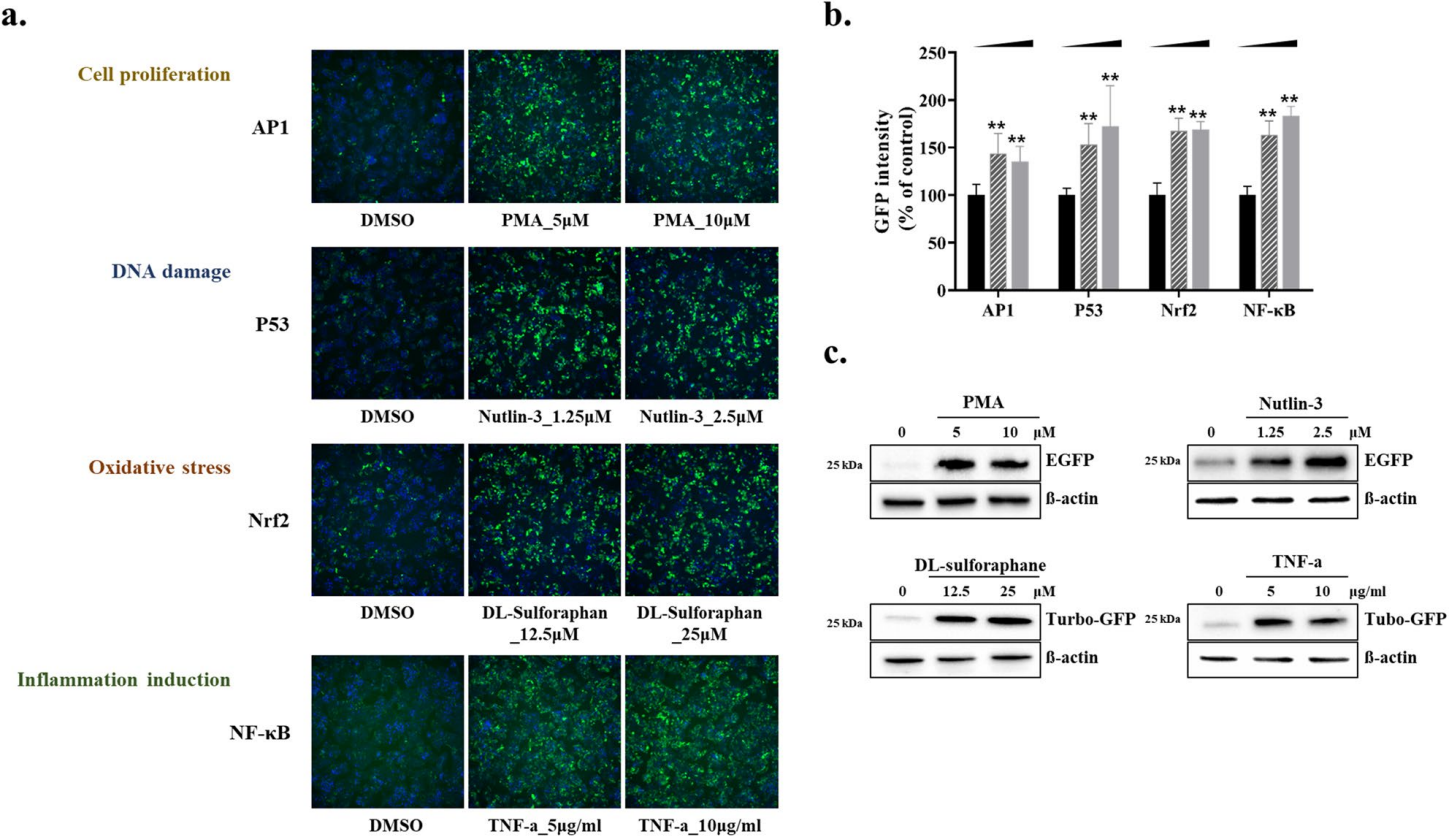
Characterization of Hepa-ToxMOA cell lines for AP1, P53, Nrf2, and NF-κB. (**a**) Cell lines were seeded at a density of 30,000 cells/well in a 96-well plate. For each positive chemical (AP1 (PMA; 5, 10 µM), P53 (Nutlin-3; 1.25, 2.5 μM), Nrf2 (DL-Sulforaphane; 12.5, 25 µM), NF-κB (TNF-α; 5, 10 µg/ml)) for cell proliferation (AP1), DNA damage (P53), Oxidative stress (Nrf2), Inflammation response (NF-κB) cellular stress-reporter cell lines, after treatment for 24 h, GFP expression was verified by 10 × image through high-content screening. (**b**) The GFP intensity value derived through HCS was quantified and shown in a graph (*p < 0.05, **p < 0.01). (**c**) Cell lines were seeded at a density of 500,000 cells/well in a 6-well plate. After treating the positive chemical with the same concentration as HCS for 24 h, western blot analysis was performed.

In this study, we established a cellular stress sensing system (Hepa-ToxMOA) that can evaluate the activation status of transcriptional factors including AP1, P53, Nrf2, and NF-κB relying on fluorescent GFP expression. These cell lines comprise the TRE for each signaling pathway and expression of GFP is induced when transcription factor is activated by cellular stress response. In order to secure the long-term stability and reproducibility of four Hepa-ToxMOA cell lines that were established, we evaluated the quality of proliferative activity, cellular morphology, and the fluorescence intensity of GFP expression according to the concentration of positive chemicals in each cell line. A high content screening (HCS) imaging system was used to confirm the change in GFP expression per cell and the optimal concentration for each positive chemical was selected for each cell line based on increases in GFP expression that did not cause reductions in cell viability. As a result, as shown in Fig. 1a,b, the GFP intensity in each cell line treated with the positive chemicals increased in each cell line compared to the control.

The level of GFP expression was also confirmed by quantifying GFP protein through western blot and it was found that the expression of GFP (HepG2-GFP-AP1, -P53, -Nrf2, and -NF-κB) increased in positive chemicals compared to the controls (Fig. 1c). The Western blot full image used for the results is shown in the Supplementary Fig. 1. Furthermore, the long-term stability of the established cell lines was confirmed by monitoring both GFP intensity and cell viability after treatment with the positive chemicals during subculture. As a result, GFP expression and cell viability were stably maintained until passage 20 (Supplementary Fig. 2). Collectively, it was confirmed that four different Hepa-ToxMOA cell lines that allow the monitoring of cellular stress was well established by showing long-term stability and reproducibility.

### Improvement of the Hepa-ToxMOA system for assessing cellular stress depending on hepatic metabolic activation

**Figure 2:**
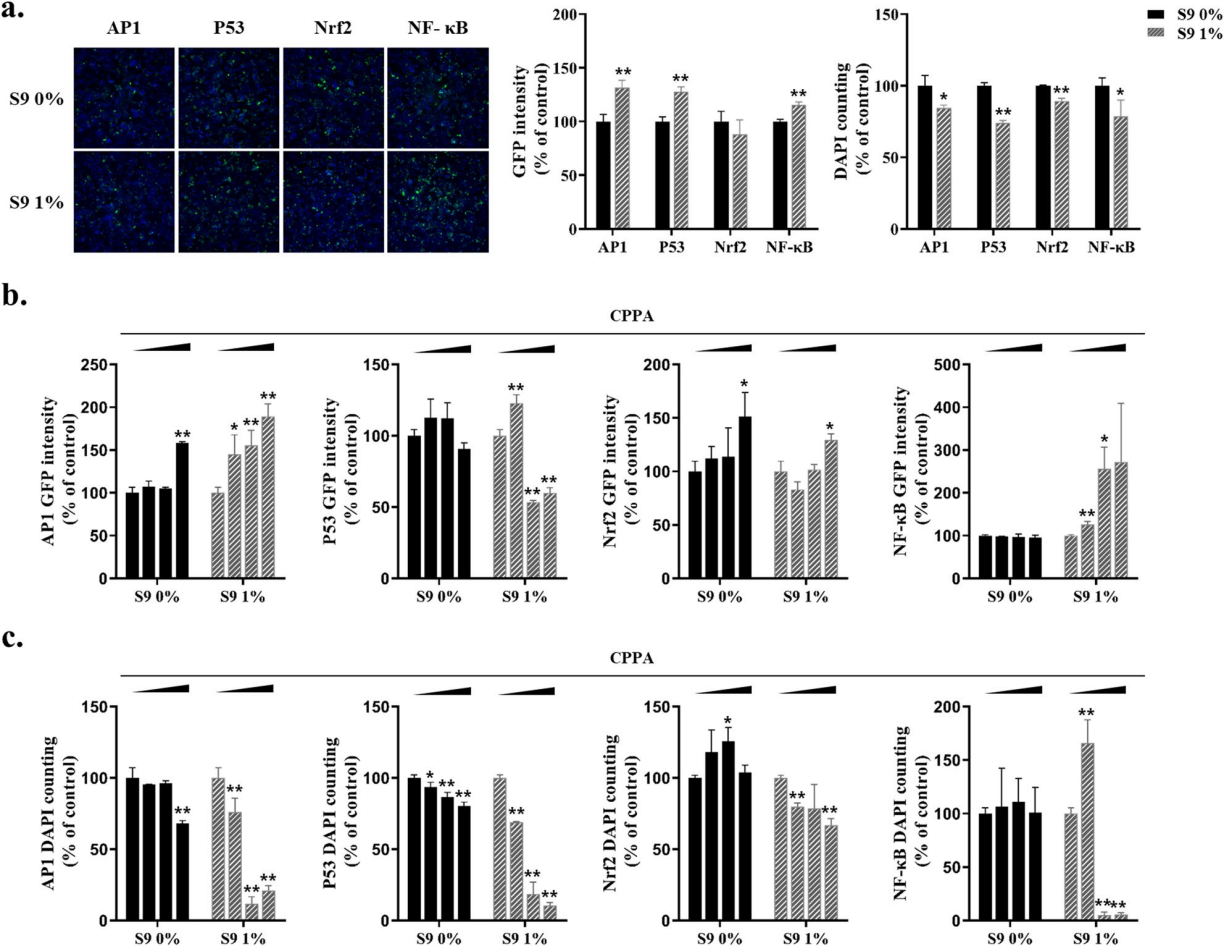
Establishment of Hepa-ToxMOA cell lines depending on metabolic activation using S9 fraction conditions. (**a**) The concentration (0% and 1%) of S9 fractions was used to treat Hepa-ToxMOA cell lines, and image analysis and GFP intensity quantification were performed through HCS (*p < 0.05, **p < 0.01). (**b**,**c**) GFP intensity and cell viability of Hepa-ToxMOA cell lines were compared after treatment for 24 h using positive control (CPPA, cyclophosphamide) 6.25, 12.5, and 25 μM according to the concentration of the S9 fraction (*p < 0.05, **p < 0.01).

To overcome the limitation of low drug metabolizing activity in HepG2 cells, we optimized the hepatic metabolic condition in the Hepa-ToxMOA cell lines using rat liver S9 fraction. Initially, we compared the GFP intensity and cell viability of the Hepa-ToxMOA cell lines treated with S9 fraction (0.25% to 1%) under basal conditions and positive control treatment (Supplementary Fig. 3). We used cyclophosphamide (CPPA) as a positive control substance, which exhibits cytotoxicity after metabolic activation by the S9 fraction. The basal level of GFP intensity and cell viability in the four Hepa-ToxMOA cell lines were partially affected by 1% S9 treatment (Fig. 2a). GFP intensity increased in a dose-dependent manner in the AP1, Nrf2, and NF-κB cell lines upon CPPA treatment, while P53 showed a tendency to increase, although not in a dose-dependent manner (Fig. 2b). Conversely, cell viability in the four Hepa-ToxMOA cell lines was significantly reduced with increasing CPPA concentration under 1% S9 treatment (Fig. 2c). These results indicate that the 1% S9 fraction consistently provides a metabolically active condition, and the optimized Hepa-ToxMOA system enables the assessment of cellular stress caused by test compounds after the drug metabolism process.

### In vitro screening of cellular stress of natural compounds using Hepa-ToxMOA system

In the preliminary study, we performed an initial screening for cytotoxic compounds among natural products using cell viability assays in human hepatocytes. From a pool of 521 natural compounds, we identified 40 substances demonstrating potential hepatotoxicity at a concentration of 10 μg/ml after 48 h of treatment. (Supplementary Table 3). To analyze the profiles of active cellular stress induced by natural compounds with or without metabolic activation, the activity of the Hepa-ToxMOA cell lines was scored based on the level of GFP intensity and cell viability of the test compounds compared to the results of the positive control (Supplementary Fig. 4).

In the Hepa-ToxMOA cell lines, natural compounds were considered positive if they induced GFP intensity to a greater degree than the positive chemical at a testing concentration that did not significantly affect cell viability. This criterion was set to ensure the reliability of GFP intensity, as it may be reduced under cytotoxic conditions. The scoring categories were as follows: 'Positive Increase (Score: 3)' indicated a cell viability of over 70% and GFP expression higher than that of the positive chemical in each cell line; 'Positive Cause (Score: 2)' indicated a cell viability of 50–70% and GFP expression higher than that of the positive chemical in each cell line; 'Possible Cause (Score: 1)' indicated a cell viability of over 50% and GFP expression lower than the positive chemical but higher than the control; and 'No Effect (Score: 0)' for substances that did not show any significant effect (Supplementary Fig. 4).

**Figure 3:**
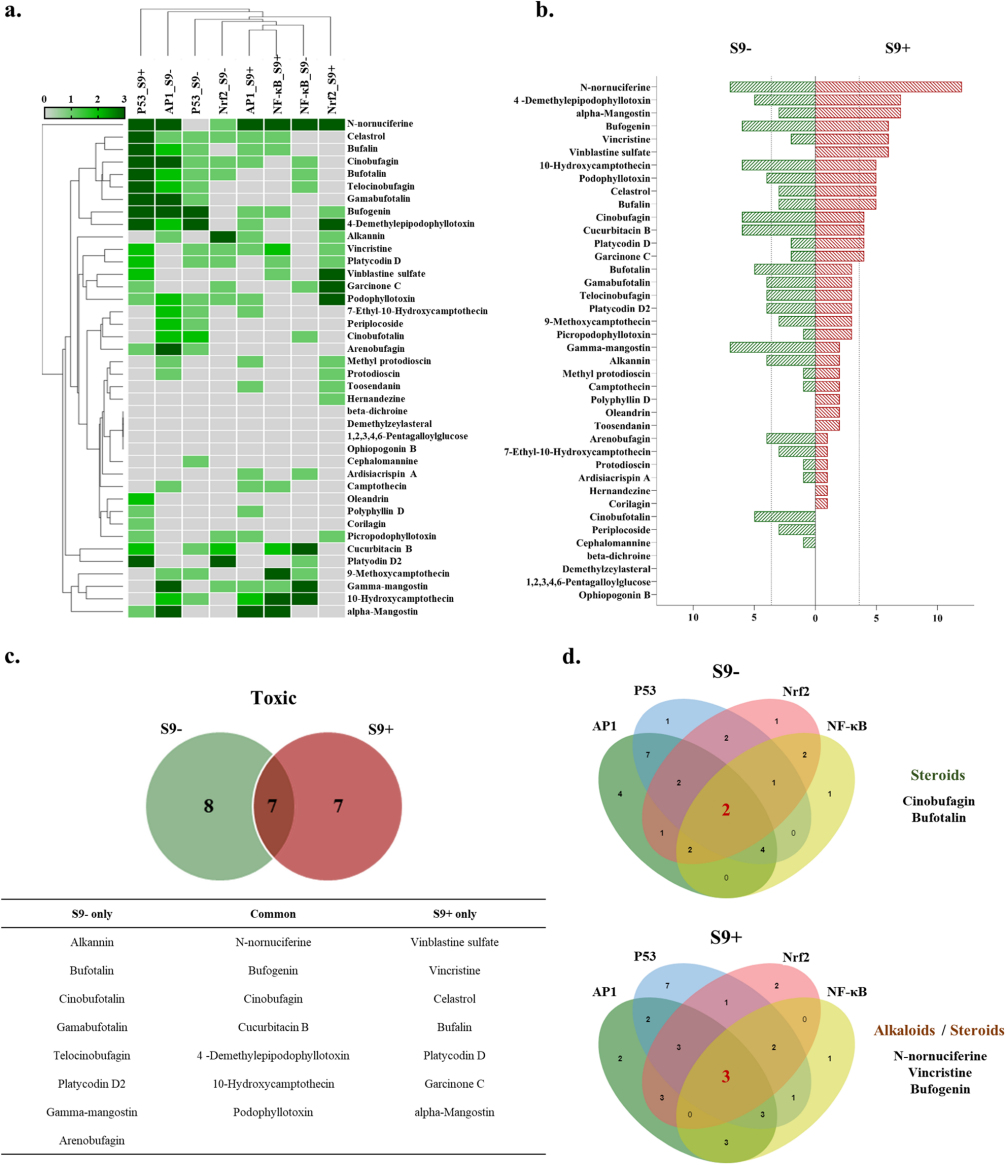
Integrated analysis of cell viability and GFP intensity expression for 40 natural products according to the non-metabolic or metabolic activity. (**a**) GFP intensity and cell viability analysis were performed after exposure of 40 natural products to four types of cellular stress-reporter cell lines for 24 h under conditions of non-metabolic or metabolic activity. Based on Supplementary Fig. 4, it was classified as a positive increase (Score 3), positive cause (Score 1 or Score 2), and no effect (Score 0). Results of a total of 40 different natural products are shown in the figures. (**b**) After scoring the expression of different toxic mechanisms in the non-metabolic or metabolic activity for 40 natural products, they were compared and analyzed with graphs and Venn diagrams (Score is a maximum of 12 points, which means a 'positive increase' in metabolic and non-metabolic conditions AP1, P53, Nrf2, and NF-κB, and of score 3 or higher, it is considered that toxicity is likely to be caused by natural product). (**c**) For the results of (**b**) above, the Venn diagram and list are shown for natural products exceeding Score 3. (**d**) In order to find natural products that are highly likely to cause toxicity under non-metabolic or metabolic activity conditions, natural products with increased GFP expression in each toxic mechanism of AP1, P53, Nrf2, and NF-κB were shown in a Venn diagram.

Based on the scoring threshold, 40 natural compounds were classified based on the activity of each cellular stress with or without metabolic activation (Fig. 3a). Heatmap and hierarchical clustering analysis revealed that AP1 and NF-κB formed closely clustered groups with metabolic activation, while AP1, P53, and Nrf2 without metabolic activation formed separate clusters. The activity of the NF-κB cell line was less affected by metabolic activation, whereas the activity of the P53 cell line was highly influenced by metabolic activation. The overall profile of active cellular stress induced by natural compounds was analyzed by summing the activity scores depending on metabolic activation (Fig. 3b). When all four cellular stresses (AP1, P53, Nrf2, and NF-κB) were commonly activated as a 'positive increase' (Score: 3), the total sum of the activity score was 12 points. As shown in Fig. 3b,c, the sum of activity scores was compared between metabolic activation and non-metabolic conditions, revealing that seven natural compounds (alpha-mangostin, vincristine, vinblastine sulfate, celastrol, bufalin, platycodin D, and garcinone C) were more active under metabolic activation than under non-metabolic conditions. On the other hand, eight natural compounds (bufotalin, gamabufotalin, telocinobufagin, platycodin D2, gamma-mangostin, alkannin, arenobufagin, and cinobufotalin) were more active without metabolic activation. Additionally, seven natural compounds (n-nornuciferine, 4-demethylepipodophyllotoxin, bufogenin, 10-hydroxycamptothecin, podophyllotoxin, cinobufagin, and cucurbitacin B) were consistently active regardless of metabolic activation. However, beta-dichroine, demethylzeylasteral, 1,2,3,4,6-pentagalloylglucose, and ophiopogonin B did not show a response in the Hepa-ToxMOA cell lines, suggesting that they may induce cytotoxicity through other signaling pathways. The Venn diagram depicts the natural compounds that increased GFP intensity in each Hepa-ToxMOA-reporter cell line of AP1, P53, Nrf2, and NF-κB (Fig. 3d). Cinobufagin and bufotalin were commonly active for all Hepa-ToxMOA cell lines without metabolic activation, while n-nornuciferine, vincristine, and bufogenin were commonly active with metabolic activation. These results demonstrate that cellular stress signaling induced by natural compounds responds differently depending on metabolic activation.

### Dose–response analysis of cellular stress induced by natural compounds depending on metabolic activation

**Figure 4:**
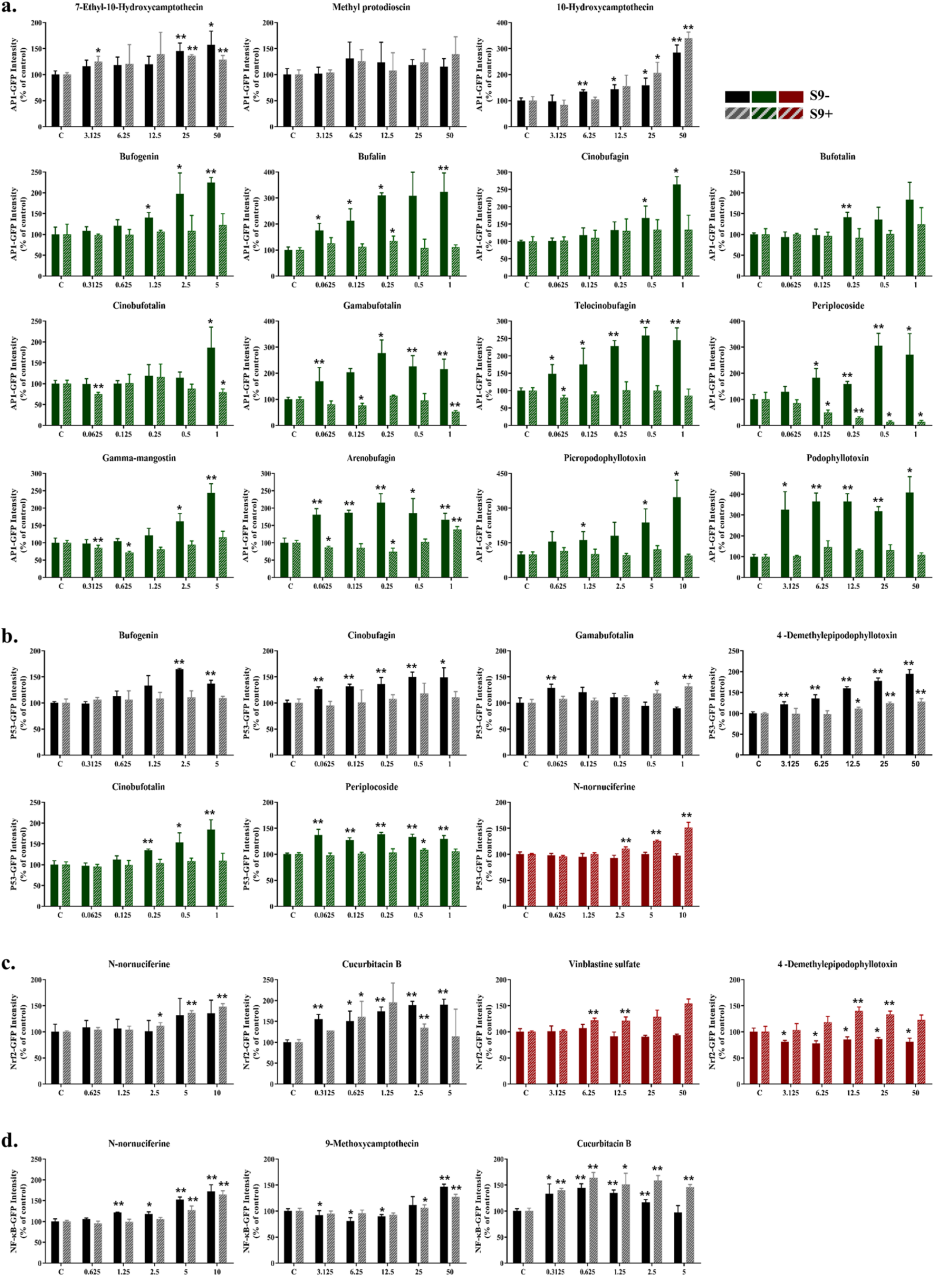
In vitro hepatotoxicity screening of 40 natural products according to the non-metabolic or metabolic activity. To perform (**a**) cell proliferation evaluation (HepG2-GFP-AP1), (**b**) DNA damage evaluation (HepG2-GFP-P53), (**c**) oxidative stress evaluation (HepG2-GFP-Nrf2), and (**d**) inflammation response evaluation (HepG2-GFP NF-κB) based on four types of cellular stress-reporter cell lines by 40 natural products under conditions of non-metabolic and metabolic activity was evaluated and analyzed. For each cellular stress-reporter cell line, natural products with increased GFP intensity regardless of non-metabolic or metabolic activity are shown in black, and natural products with increased intensity only under conditions of non-metabolic activity are shown in green. Natural products with increased only under conditions of metabolic activity are shown in red (Full bar mean S9- and hatched bar mean S9 +) (*p < 0.05, **p < 0.01).

Dose–response analysis of the natural compounds selected in Fig. 3b was conducted in detail using the Hepa-ToxMOA cell line (Fig. 4). The GFP intensity was compared depending on metabolic activation in each cell line, and the natural compounds were classified into three groups based on their activity patterns: commonly active (gray box), S9-specific active (green box), and S9 + specific active (red box) groups based on GFP expression patterns (Fig. 4).

In the AP1 cell line, which is used to evaluate cell proliferation mediated by natural compounds, 7-ethyl-10-hydroxycamptothecin, methyl protodioscin, and 10-hydroxycamptothecin showed increased GFP intensity regardless of metabolic activation. A total of 12 natural compounds (bufogenin, bufalin, cinobufagin, bufotalin, cinobufotalin, gamabufotalin, telocinobufagin, periplocoside, gamma-mangostin, arenobufagin, picropodophyllotoxin, and podophyllotoxin) specifically induced GFP expression in the AP1 cell line without metabolic activation, and their activity did not respond under conditions of metabolic activation (Fig. 4a).

In the P53 cell line, which is used to evaluate DNA damage induced by natural compounds, GFP expression was increased by bufogenin, cinobufagin, gamabufotalin, and 4-demethylepipodophyllotoxin regardless of metabolic activation. N-nornuciferine from the alkaloids family showed no change in GFP expression without metabolic activation, but GFP expression in the P53 cell line increased notably, up to 1.5-fold, under metabolic activation conditions. On the other hand, P53 was specifically activated by cinobufotalin and periplocoside from the steroids family without metabolic activation, but there was no change in the expression of GFP under metabolic activation conditions (Fig. 4b).

As shown in Fig. 4c, the Nrf2 cell line was used to evaluate oxidative stress induced by natural compounds. N-nornuciferine and cucurbitacin B induced GFP expression in the Nrf2 cell line regardless of metabolic activation. Vinblastine sulfate and 4-demethylepipodophyllotoxin showed no change in GFP expression in the Nrf2 cell line without metabolic activation, but Nrf2 was specifically activated under metabolic activation. The NF-κB cell line was used to evaluate induction of inflammation by natural compounds. N-nornuciferine, 9-methoxycamptothecin, and cucurbitacin B commonly induced increased GFP expression regardless of metabolic activation (Fig. 4d). These results demonstrate that natural compounds induce cytotoxicity in HepG2 cells through different cellular stress responses depending on metabolic activation.

## Discussion

The present study focused on the establishment and optimization of a cellular stress sensing system called Hepa-ToxMOA for the screening of natural compounds and the analysis of their toxicity mechanisms. To select representative transcription factors related to cellular stress for the screening of natural products, we conducted an analysis of open-source databases focusing on natural products^24^ and toxic mechanisms (EPA's Tox 21 project and PubChem database). This analysis led us to identify four transcription factors (AP1, P53, Nrf2, and NF-κB) that are involved in regulating cellular stress induced by various natural compounds, including alkaloids and di-/tri-terpenoids.

AP1 and Nrf2 are representative toxicity mechanisms that can induce apoptosis and cell hyperphagia through toxicity-inducing factors^25,26^. AP1 is a transcription factor that regulates gene expression in response to various stimuli, including cytokines, growth factors, and stress. It controls cellular processes such as differentiation, proliferation, and apoptosis^27,28^. Nrf2, on the other hand, is a transcription factor that regulates antioxidant genes by binding to antioxidant response elements. It plays a crucial role in the response to oxidative stress and is associated with the development of liver diseases like drug-induced liver injury, fatty liver disease, and liver fibrosis^29^, and which is a major cause of development such as drug-induced liver injury, fatty liver disease, and liver fibrosis^30^. Moreover, recent studies on adverse outcome pathways (AOP) related to hepatotoxicity have identified the Nrf2 activity stage as a key event in terms of molecular aspects for aryl hydrocarbon receptor (AHR) activity^31^. P53 is a transcription factor that binds to more than 4000 sites in the genome and regulates the expression of over 500 genes. It affects various cellular processes, including glucose, lipid, and amino acid metabolism, oxidative phosphorylation, reactive oxygen species (ROS) generation, and growth factor signaling^32^. P53 can be induced by perturbations of oxygen tension, nutrient availability, or redox state, such as DNA damage^33^. Its induction can regulate cell metabolism differently by inducing autophagy and apoptosis, depending on the regulation of the PI3K/AKT/mTOR pathway and ROS induction. It also acts as a general stress sensor, important for reducing cell proliferation, altering cell metabolism, and inhibiting survival in response to viral infection, starvation, or oxidative stress^32^. Furthermore, NF-κB is a key transcriptional regulator of the inflammatory response in the liver and is required for hepatocyte survival and liver homeostasis. It influences the development of chronic liver injury, fibrosis, and hepatocellular carcinoma^34,35^. Therefore, the cellular stress-reporter cell lines established based on these mechanisms can serve as a primary monitoring and evaluation system for toxicity, directly impacting cell stimulation and damage.

The metabolic activation of natural products is mediated by gut bacteria, particularly cytochrome P450, which binds to glutathione or macromolecules and generates reactive metabolites that form glutathione, protein, and DNA adducts^6^. Thus, when evaluating hepatotoxicity using cell lines, considering the activation condition of the cell line based on metabolic activity is crucial. Different toxic responses may indicate metabolic-dependent toxicity^36^. Traditionally, the HepG2 cell line has been widely utilized for high-throughput screening of hepatotoxicity due to its ability to exhibit crucial hepatocyte characteristics like albumin production^37^. However, HepG2 cells lack essential metabolic competence, including the majority of CYP450 enzymes, necessary for faithfully replicating in vivo conditions. We compared the expression levels of key CYP isoforms in HepG2 and HepG2-Nrf2, in comparison to primary human hepatocytes (PHH), which are considered a gold standard with high drug metabolism activity (Supplementary Fig. 5). It was found that the levels of *CYP1A1*, *CYP3A4*, *CYP2E1*, *CYP2C19*, and *CYP2D6* were significantly lower in both HepG2 cells and the HepG2-Nrf2 cell line compared to PHH (Supplementary Fig. 5). Several models, such as a recombinant CYP expression system, microsomes, have been employed to enhance drug metabolism capabilities, although the in vitro metabolic activation system cannot fully replicate the intricate drug metabolism observed in in vivo testing^38^. Among these systems, recombinant CYPs and microsomes have limitations in representing phase 2 enzymes. Conversely, the S9 fraction, consisting of a blend of microsomes and cytosol, encompasses a broad spectrum of Phase I CYP isoforms and Phase II drug-metabolizing enzymes, resembling hepatocytes^38,39^. In this study, we optimized the Hepa-ToxMOA cell lines derived from HepG2 to augment metabolic activity using S9 fractions. This study confirms the enhancement of drug metabolism activity in the Hepa-ToxMOA system by using a positive compound, cyclophosphamide (CPPA), which exhibits cytotoxicity after metabolic activation by the S9 fraction. The higher GFP intensity of CPPA-treated Hepa-ToxMOA cell lines supplemented with the S9 fractions compared to those without the S9 fractions indicates that the Hepa-ToxMOA system can compensate for the lack of CYP expression in HepG2 cell lines. In addition, S9 fractions provide benefits for high-throughput screening (HTS) methods, and we have refined the S9 treatment conditions in the Hepa-ToxMOA system that may be used for in vitro* s*creening. Our research contributes to a better understanding of the metabolic activity of natural products and its implications for hepatotoxicity screening.

There is substantial evidence supporting the notion that natural products can either increase toxicity or provide protection due to their metabolic activity. For instance, vinblastine has been shown to induce CYP3A4 via the activation of NR1I2. Since vinblastine is a substrate of CYP3A4, an overdose or long-term administration of the drug can accelerate its metabolism. Combination administration with other drugs may also alter the efficacy or toxicity of vinblastine due to possible interactions^40^. Similarly, cinobufagin and bufotalin have been identified as substrates for both CYP3A4 and CYP3A5^41^. Bufotalin, in particular, is a natural product that has received attention as an anticancer drug, and research on its metabolic pathways in humans is currently being conducted. It has been observed that bufotalin is metabolized by CYP3A to 5ß-hydroxy-bufotalin, confirming its adherence to autoactivation kinetics and substrate inhibition kinetics^42^. On the other hand, alkannin has demonstrated hepatoprotective effects on liver injury in diabetic C57BL/KsJ-db/db mice, and further characterization of its hepatoprotective effects has been carried out in damaged HepG2 cells^43^. These findings support our results indicating reduced toxicity in Hepa-ToxMOA cell lines under metabolic activity conditions. Additionally, gamma-mangostin has been found to attenuate fasting blood glucose levels in diabetic mice without exhibiting hepatotoxicity in a study on the treatment of diabetes mellitus^44^. Furthermore, it has been shown to alleviate liver fibrosis^45^. These observations explain the significant decrease in toxicity of gamma-mangostin under metabolic activity conditions. A comprehensive analysis of these results underscores the importance of evaluating drug metabolism for the assessment of hepatotoxicity using cell lines.

In conclusion, the results obtained from the Hepa-ToxMOA system highlight its importance as a screening tool for assessing the cellular stress induced by natural compounds. The ability of the system to evaluate the activation status of transcription factors involved in cellular stress response pathways provides valuable information about the toxicity mechanisms of natural compounds. The study demonstrated the system's long-term stability, reproducibility, and optimization for metabolic activation, further enhancing its utility in toxicity screening. Overall, the Hepa-ToxMOA system offers a valuable platform for future studies investigating the cytotoxicity and stress responses induced by natural compounds, ultimately contributing to the development of safer and more effective therapeutic agents.

### Supplementary Information

Supplementary Figures.

Supplementary Tables.

## Data Availability

The datasets used and/or analyzed during the current study available from the corresponding author on reasonable request.
