# Supplementary material for: Hepa-ToxMOA: a pathway-screening method for evaluating cellular stress and hepatic metabolic-dependent toxicity of natural products
Source: Sci Rep. 2024 Feb 21;14:4319. doi: 10.1038/s41598-024-54634-4 (PMC10881971; doi:10.1038/s41598-024-54634-4)
Supplement: Supplementary file 1 — Supplementary Figures. [file 41598_2024_54634_MOESM1_ESM.pdf]

Supplementary Figure 1.

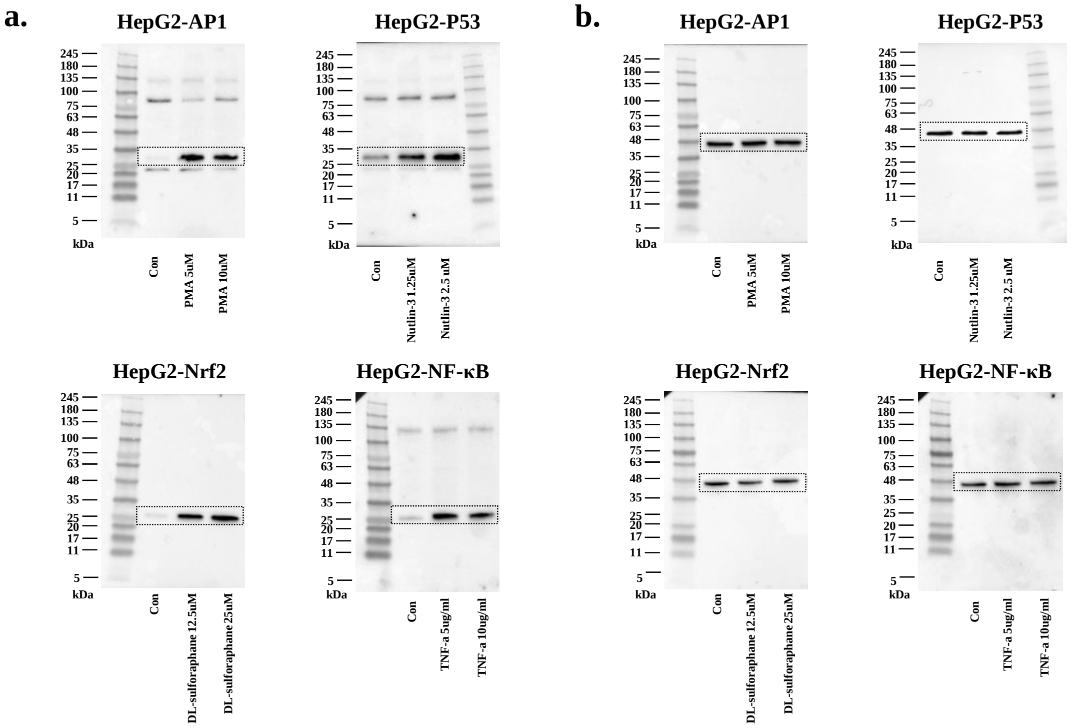

Supplementary Figure 1. Uncropped full-length picture of western blotting membranes. Uncropped full-length pictures of western blotting membranes presented in the main Fig. 1c. (a) Anti-EGFP Anti-TurboGFP GFP western blot results for HepG2-AP1, P53, Nrf2, and NF-κB. (b) ß-actin western blot results for HepG2-AP1, P53, Nrf2, and NF-κB.

Supplementary Figure 2.

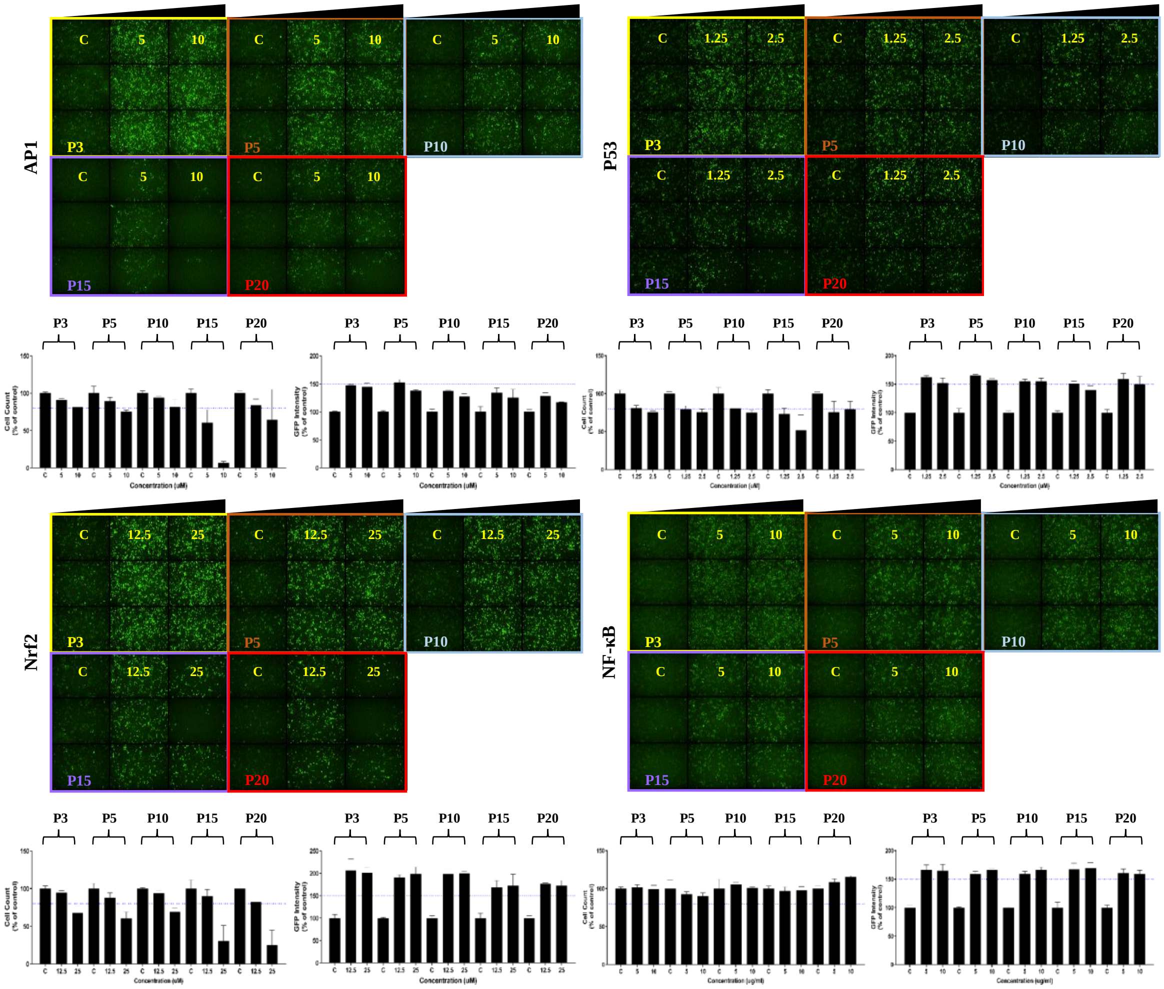

Supplementary Figure 2. Stabilization and reproducibility of HepG2-GFP-AP1, HepG2-GFP-P53, HepG2-GFP-Nrf2, HepG2-GFP-NF-κB cell lines.

The data on the cell proliferation, cell morphology, and GFP intensity of positive chemicals according to the increase in the subculture (final passage 20) of the four types of cellular stress-reporter cell lines were analyzed and are shown in images and graphs.

Supplementary Figure 3.

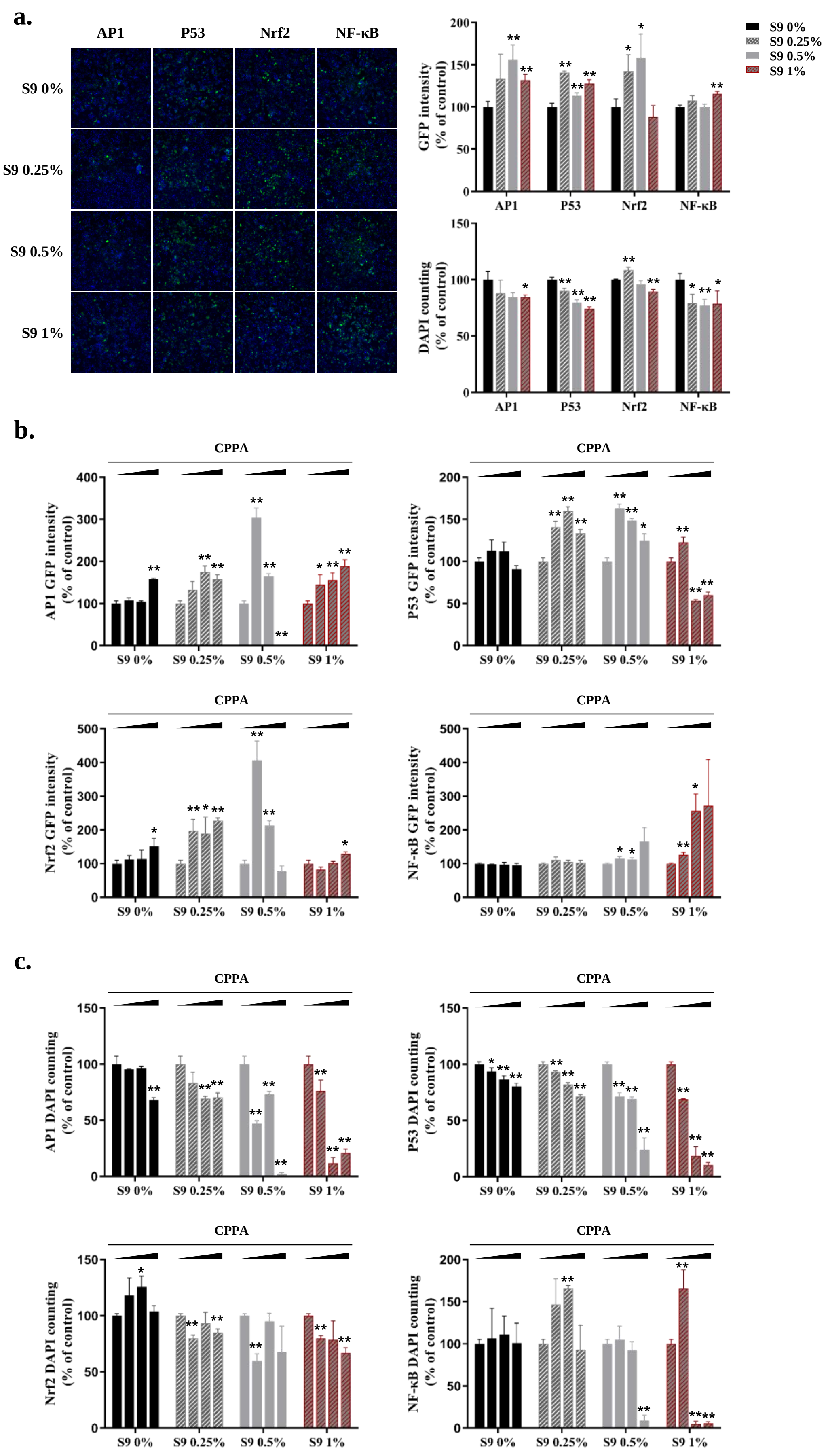

Supplementary Figure 3. Optimization of S9 fraction conditions to increase drug metabolism efficacy of Hepa-ToxMOA cell lines.

(a) The concentration (0%, 0.25%, 0.5%, and 1%) of S9 fractions was used to treat four types of cellular stress-reporter cell lines, and image analysis and GFP intensity quantification were performed through HCS (*$p<0.05$, **$p<0.01$). (b)(c) GFP intensity and cell viability of Hepa-ToxMOA cell lines were compared after treatment for 24 hours using positive control (CPPA, cyclophosphamide) 6.25, 12.5, and 25 μM according to the concentration of the S9 fraction (*$p<0.05$, **$p<0.01$).

Supplementary Figure 4.

	GFP Intensity (High increase)	GFP Intensity (Low increase)	GFP Intensity (No change)
Cell Viability (70-100%)	Positive increase (3)	Positive cause (1)	No effect (0)
Cell Viability (50-70%)	Positive cause (2)	Positive cause (1)	No effect (0)
Cell Viability (0-50%)	No effect (0)	No effect (0)	No effect (0)

Supplementary Figure 4. Classification of cell viability and GFP intensity of Hepa-ToxMOA cell lines.

'Positive increase (Score: 3)' means a cell viability of over 70% and GFP expression is higher than that of positive chemical in each cell line, 'Positive cause (Score: 2)' means a cell viability of 50%–70% and GFP expression is higher than that of positive chemical in each cell line, 'Positive cause (Score: 1)' means a cell viability of over 50% and GFP expression lower than positive chemical but higher than control, and other substances have 'No effect (Score: 0)', the steps from 0 to 3 are indicated in green.

Supplementary Figure 5.

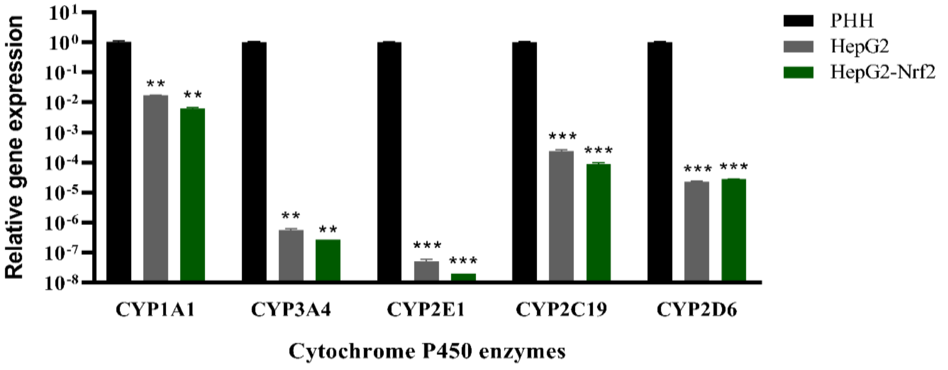

Supplementary Figure 5. The expression level of cytochrome P450 (CYP) isoforms in human hepatocytes using quantitative real-time RT-PCR

The mRNA expression levels of cytochrome *CYP1A1*, *CYP3A4*, *CYP2E1*, *CYP2C19* and *CYP2D6* were analyzed in primary human hepatocytes, HepG2 and HepG2-Nrf2 cell line from Hepa-ToxMOA. Primary human hepatocytes (Corning, NY, USA, Donor No. 409) were used as the reference cell line for CYP expression. The relative fold change in expression level was presented compared to that of PHH. *$p<0.05$, **$p<0.01$, ***$p<0.001$. Total RNA (1 μg) was reverse transcribed from each cell lines using a high-capacity cDNA reverse transcription kit (Applied Biosystems, CA, USA) according to the manufacturer's instructions. Quantitative real-time RT-PCR was performed in a 20 μl reaction volume containing 1 μl (5 pM) forward and reverse specific primers, 10.2 μl of GoTaq® qPCR Master Mix (Promega, WI, USA), 5 μl of cDNA and 3.8 μl of nucleasefree water. The cDNA was amplified using a StepOnePlusTM Real-Time PCR System (Applied Biosystems) following the manufacturer’s protocol. The 18S ribosomal RNA primers were used as an internal control. Primer sequences for all genes investigated are as follows: *CYP1A1* (Forward: TCTGTGCCATTTGCTTTGGC, Reverse: AGGCATTCAGGGAAGGGTTG), *CYP3A4* (Forward: CCGAGTGGATTTCCTTCAGCTG, Reverse: TGCTCGTGGTTTCATAGCCAGC), *CYP2E1* (Forward: GAGCACCATCAATCTCTGGACC, Reverse: CACGGTGATACCGTCCATTGTG), *CYP2C19* (Forward: ACCAGGGTTTAATCTTTTTCAGC, Reverse: GCTTTGGAGATGACATGAGGC), *CYP2D6* (Forward: CGCATCCCTAAGGGAACGA, Reverse: TTCCAGACGGCCTCATCCT) and 18S rRNA (Forward: ACCCGTTGAACCCCATTCGTGA, Reverse: GCCTCACTAAACCATCCAATCGG).
