# Supplementary material for: Hepa-ToxMOA: a pathway-screening method for evaluating cellular stress and hepatic metabolic-dependent toxicity of natural products
Source: Sci Rep. 2024 Feb 21;14:4319. doi: 10.1038/s41598-024-54634-4 (PMC10881971; doi:10.1038/s41598-024-54634-4)
Supplement: Supplementary file 2 — Supplementary Tables. [file 41598_2024_54634_MOESM2_ESM.docx]

**Supplementary Table 1. Lentiviral vectors information for establishing Hepa-ToxMOA cell lines.**

| **Product** | **Manufacturer** | **Catalog No.** | **Sequence motif** | **GFP** |
| --- | --- | --- | --- | --- |
| pGreenFire1^TM^-AP1(Plasmid)+EF1-Puro | System biosciences | TR201PA-P | 5'-GAATTCGGTGACTCAGTGACTGAGTCAGATAGGTGACTCAGTGACTGAGTCAGATAGGTGACTCAGTGACTGAGTCAGATAGGTGACTCAGTGACTGAGTCAGATAACTAGT | copGFP |
| pGreenFire1^TM^- P53-GF-EF1-Puro (Plasmid) | System biosciences | TR200PA-P | 5'-GAATTCGGACATGCCCGGGCATGTCCCCAGGGACATGCCCGGGCATGTCCCCAGAGACATGTCCAGACATGTCCCCAGGAACATGTCCCAACATGTTGTCCAGGAGACATGTCCAGACATGTCCCCAGGAACATGTCCCAACATGTTGTACTAGT | copGFP |
| ARE-GFP(Puro) Lentivirus | AMSBIO | LV981-P | 5'- TCACAGTGACTCAGCAAAATT | e-GFP |
| NF-kB-GFP(Puro) Lentivirus | AMSBIO | LV965-P | 5'- GGGACTTTCC | e-GFP |

**Supplementary Table 2. Information on all reagents used in the experiment.**

| Materials and methods | **Product** | **Manufacturer** | **Catalog No.** |
| --- | --- | --- | --- |
| 2.1. Cell culture | HepG2 cell lines | ATCC | HB-8065 |
|  | Dulbecco’s Modified Eagle’s Medium (DMEM) | Lonza | BE12-614F |
|  | Fetal Bovine Serum | Gibco | 16000-044 |
|  | Penicillin/Streptomycin | Lonza | DE17-602E |
|  | pPACKH1TM Lentivector Packaging Kit | System biosciences | LV500A-1 |
|  | Lenti-X Concentration | Clontech | PT4421-2 |
| 2.2 Natural Products | Dimethyl sulfoxide (DMSO) | Sigma-Aldrich | D2650 |
| 2.3. S9 fraction | Rat Liver S9 | Molecular Toxicology | 11-01L.2 |
|  | Cofactor I | Oriental Yeast | 309-50611 |
| 2.4. Western blotting | Pierce BCA protein assay kit | Thermo | 23227 |
|  | 10X TBS | BioRad | 1706435 |
|  | Tween 20 | BIOPURE | 8995T |
|  | Skim milk | BD | 232100 |
|  | Anti-TurboGFP(d) antibody | Evrogen | AB513 |
|  | Anti-EGFP antibody | Abcam | Ab290 or Ab184601 |
| 2.5. High-Contents Screening | Hoechst 33342 | Thermo | H3570 |
|  | Calcein-AM | Invitrogen | C3100MP |
|  | DPBS (without calcium chloride, without magnesium chloride) | WELGENE | LB 001-02 |
|  | DPBS (with calcium chloride, with magnesium chloride) | WELGENE | LB 001-01 |

**Supplementary Table 3. Identification of potentially hepatotoxic compounds from a pool of 521 natural compounds.**

|  | Class I^a^ | Class II^b^ | Class III^c^ | Total |
| --- | --- | --- | --- | --- |
| No. of  compounds | 40 (7.7%) | 74 (14.2%) | 407 (78.1%) | 521 |

^a-c^ Class I: cytotoxic at 10 μg/ml; Class II: cytotoxic at 100 μg/ml, but not at 10 μg/ml; Class III: non-cytotoxic at 10 and 100 μg/ml. 40 natural compounds for Class I were selected for the further experiments in this study.

A total of 521 compounds (purity >98% HPLC) were purchased from Chengdu Alfa Biotechnology Co., Ltd (Chengdu, China). These compounds were dissolved in autoclaved water or dimethyl sulfoxide (DMSO; Sigma-Aldrich, USA), and the final concentration of DMSO in the medium was maintained at 0.5% (v/v). The Chang liver cells were cultured in Dulbecco’s modified Eagle’s medium (DMEM) containing 10% heat-inactivated fetal bovine serum (FBS) obtained from HyClone Laboratories (Logan, UT, USA) and 1% penicillin–streptomycin (Gibco Biotechnology, Waltham, MA, USA). Cells were incubated at 37 °C under 5% CO_2_, seeded into 96-well microculture plates (7 x 10^3^ cells/well) for 24 h, and treated with 521 compounds (10 and 100 μg/ml) for 48 hr. Following treatment, 20 μL of a 5 mg/mL solution of 3-(4,5-dimethylthiazol-2-yl)-2,5-diphenyltetrazolium bromide (MTT) in phosphate-buffered saline (PBS) was added to each well, and the plates were incubated for 4 h in the dark. The supernatant was removed, and the insoluble formazan crystals were dissolved in 200 μL of DMSO. Absorbance was measured at 595 nm using a Thermomax microplate reader (Molecular Devices, San Jose, CA, USA). Cytotoxicity measured by MTT assay was determined using the statistical left normal distribution method (3 sigma limit) at concentrations of 10 and 100 μg/mL. 40 natural compounds for Class I were selected for the further experiments in this study.

**Supplementary Table 4. Information on positive chemicals and 40 natural compounds including alkaloids, quinones, steroids, triterpenoids, xanthone, and diterpenoids.**

|  | **Product** | **Manufacturer** | **Catalog No.** | **MW** | **Cas #** | **Final concentration** |
| --- | --- | --- | --- | --- | --- | --- |
| Positive chemical | PMA | Sigma-Aldrich | P1585 | 616.83 | 16561-29-8 | 5 μM, 10 μM |
|  | Nutlin-3 | Sigma-Aldrich | N6287 | 581.49 | 548472-68-0 | 1.25 μM, 2.5 μM |
|  | DL-sulforaphane | Sigma-Aldrich | S4441 | 177.29 | 4478-93-7 | 12.5 μM, 25 μM |
|  | TNF-a | Sigma-Aldrich | SRP3177 | - | 3483-12-3 | 5 μg/ml, 10 μg/ml |
| Natural products | beta-febrifugine  (beta-dichroine) | Tauto Biotech | E-0677 | 301.344 | 24159-07-7 | 50 μg/ml |
|  | N-nornuciferine | Tauto Biotech | E-2038 | 281.4 | 4846-19-9 | 10 μg/ml |
|  | Vinblastine sulfate | Tauto Biotech | E-0181 | 909.0526 | 143-67-9 | 50 μg/ml |
|  | 9-Methoxycamptothecin | Tauto Biotech | E-3013 | 378.38 | 39026-92-1 | 50 μg/ml |
|  | 7-Ethyl-10-Hydroxycamptothecin | Tauto Biotech | E-0291 | 392.4046 | 86639-52-3 | 50 μg/ml |
|  | Vincristine | ChemFaces | CFN98589 | 824.96 | 57-22-7 | 50 μg/ml |
|  | Celastrol | Tauto Biotech | E-0270 | 450.60962 | 34157-83-0 | 1 μg/ml |
|  | Alkannin | Tauto Biotech | E-0403 | 288.29524 | 517-89-5 | 1 μg/ml |
|  | Bufogenin | Tauto Biotech | E-0107 | 384.50848 | 465-39-4 | 5 μg/ml |
|  | Polyphyllin D | Tauto Biotech | E-0285 | 855.02 | 50773-41-6 | 5 μg/ml |
|  | Methyl protodioscin | Tauto Biotech | E-0601 | 410.5 | 54522-52-0 | 50 μg/ml |
|  | Bufalin | Tauto Biotech | E-0531 | 386.52 | 465-21-4 | 1 μg/ml |
|  | Protodioscin | Tauto Biotech | E-0163 | 1049.21 | 55056-80-9 | 50 μg/ml |
|  | Cinobufagin | Tauto Biotech | E-0487 | 442.54 | 470-37-1 | 1 μg/ml |
|  | Bufotalin | Tauto Biotech | E-0532 | 444.56 | 471-95-4 | 1 μg/ml |
|  | Cinobufotalin | Tauto Biotech | E-2492 | 458.54 | 1108-68-5 | 1 μg/ml |
|  | Gamabufotalin | Tauto Biotech | E-0929 | 402.52 | 465-11-2 | 1 μg/ml |
|  | Telocinobufagin | Tauto Biotech | E-3075 | 402.52 | 472-26-4 | 1 μg/ml |
|  | Periplocoside | Tauto Biotech | E-0426 | 696.825 | 13137-64-9 | 1 μg/ml |
|  | Oleandrin | Tauto Biotech | E-0814 | 576.72 | 465-16-7 | 1 μg/ml |
|  | Demethylzeylasteral | Tauto Biotech | E-0797 | 480.59 | 107316-88-1 | 10 μg/ml |
|  | Cucurbitacin B | Tauto Biotech | E-0442 | 558.7 | 6199-67-3 | 5 μg/ml |
|  | Platycodin D2 | Tauto Biotech | E-2519 | 1387.46 | 66663-90-9 | 10 μg/ml |
|  | Ardisiacrispin A | Tauto Biotech | E-1213 | 1061.22 | 23643-61-0 | 1 μg/ml |
|  | Toosendanin | Tauto Biotech | E-0634 | 574.62 | 58812-37-6 | 50 μg/ml |
|  | Platycodin D | Tauto Biotech | E-0660 | 1225.34068 | 58479-68-8 | 10 μg/ml |
|  | Garcinone C | Tauto Biotech | E-0780 | 414.45 | 76996-27-5 | 5 μg/ml |
|  | Gamma-mangostin | Tauto Biotech | E-0786 | 396.43 | 31271-07-5 | 5 μg/ml |
|  | 1,2,3,4,6-Pentagalloylglucose | Tauto Biotech | E-1144 | 940.68 | 14937-32-7 | 10 μg/ml |
|  | Arenobufagin | Tauto Biotech | E-0947 | 416 | 464-74-4 | 1 μg/ml |
|  | Ophiopogonin B | ChemFaces | CFN98555 | 722.91 | 38971-41-4 | 10 μg/ml |
|  | Hernandezine | Phytounique | BP0721 | 652.788 | 6681-13-6 | 10 μg/ml |
|  | 4-Demethylepipodophyllotoxin | Biorbyt | orb320552 | 400.38 | 6559-91-7 | 50 μg/ml |
|  | 10-Hydroxycamptothecin | Biorbyt | orb390029 | 364.35 | 19685-09-7 | 50 μg/ml |
|  | alpha-Mangostin | Biorbyt | orb593733 | 410.46 | 6147-11-01 | 5 μg/ml |
|  | Camptothecin | Biorbyt | orb259154 | 348.358 | 7689-03-04 | 10 μg/ml |
|  | Cephalomannine | Biorbyt | orb105502 | 831.912 | 71610-00-9 | 50 μg/ml |
|  | Corilagin | Biorbyt | orb593838 | 634.46 | 23094-69-1 | 50 μg/ml |
|  | Picropodophyllotoxin | Biorbyt | orb259440 | 414.41 | 477-47-4 | 10 μg/ml |
|  | Podophyllotoxin | Biorbyt | orb322668 | 414.41 | 518-28-5 | 50 μg/ml |
